# Discrepancies in Lymphoma Diagnosis Over the Years: A 13-Year Experience in a Tertiary Center

**DOI:** 10.4274/tjh.2016.0344

**Published:** 2017-03-01

**Authors:** Neval Özkaya, Nuray Başsüllü, Ahu Senem Demiröz, Nükhet Tüzüner

**Affiliations:** 1 İstanbul University Cerrahpaşa Faculty of Medicine, Department of Pathology, İstanbul, Turkey; 2 İstanbul Bilim University Faculty of Medicine, Department of Pathology, İstanbul, Turkey; 3 The current affiliation for N.Ö. is Department of Pathology, Memorial Sloan Kettering Cancer Center, New York, USA

**Keywords:** Diagnosis of lymphoma, World Health Organization lymphoma classification, Discrepancies in diagnosis, Hematopathology

## Abstract

**Objective:**

In the past, accurate diagnosis of lymphoma was challenging since there were multiple competing classification systems that caused confusion and debate. After establishment of the World Health Organization lymphoma classification, lymphomas still remain a diagnostic challenge among general pathologists. The purpose of this study was to examine whether the discordance among centers has declined over the years.

**Materials and Methods:**

All lymphoma or lymphoma-suspected specimens that had been sent to the Cerrahpaşa Faculty of Medicine between 2000 and 2013 for a second opinion were deemed eligible. To evaluate the change in the discrepancy rates over time we compared the rates of revision between 2000-2008 and 2009-2013.

**Results:**

A total of 1824 patients in two time periods met the inclusion criteria. The overall discordance rate was 45.6%. This rate showed significant variations between different histologic subtypes. Discordance rates also varied significantly over time and decreased from 51.3% in 2000-2008 to 38.7% in 2009-2013 (p<0.0001).

**Conclusion:**

The high discordance rate, especially in the second period, indicates the need for easily accessible hematopathology consultation centers.

## Abstract (tr)

**Amaç:**

Geçmiş yıllarda, farklı birçok lenfoma sınıflandırma sisteminin yarattığı karmaşa nedeniyle doğru bir lenfoma tanısına ulaşmak tüm patologlar için uğraşı gerektirmekteydi. Dünya Sağlık Örgütü’nün lenfoma sınıflandırmasıyla birlikte lenfoma tanı ve sınıflamasına büyük ölçüde açıklık getirilmesine rağmen genel patologlar için doğru lenfoma tanısı hala bir uğraşı nedenidir. Bu çalışmanın amacı değişik merkezlerdeki tanı farklılıklarının zaman içinde azalıp azalmadığını araştırmaktır.

**Gereç ve Yöntemler:**

2000-2013 yılları arasında Cerrahpaşa Tıp Fakültesi, Patoloji Anabilim Dalı’na lenfoma tanı ve/veya şüphesi ile konsültasyona gönderilen tüm olgular çalışma kapsamına alındı. Lenfoma tanısında, konsültasyon merkezi ile tutarsızlık oranlarındaki değişimleri değerlendirmek amacı ile 2000-2008 ve 2009-2013 yılları arasındaki tanı tutarsızlık oranları karşılaştırıldı.

**Bulgular:**

Çalışma kapsamına giren 1824 hastada genel uyumsuzluk oranı %45,6 olup değişik histolojik alt tipler arasında önemli farklılıklar saptandı. Tanılar arasındaki tutarsızlık oranları 2000-2008 yılları arasında %51,3 iken 2009-2013 arasında %38,7’ye gerileyerek zaman içinde anlamlı ölçüde azalma gösterdi (p<0,0001) ancak hala çok yüksekti.

**Sonuç:**

Özellikle 2009-2013 yılları arasında hala tanılar arasındaki tutarsızlık oranlarının yüksek oluşu, genel patologlar ve hematolog/onkologların kolayca ulaşabilecekleri hematopatoloji konsültasyon merkezlerinin önemini vurgulamaktadır.

## INTRODUCTION

Accurate histologic diagnosis is the most crucial step for the appropriate management of patients with lymphoma. In the past this was challenging since there were numerous competing classification systems, which caused conflict and discussion [1,2]. In 2000, a new unified diagnostic classification system was recommended by the World Health Organization (WHO) based on the Revised European-American Classification of Lymphoid Neoplasms (REAL) with an emphasis on the importance of morphologic, immunophenotypic, molecular, and genetic features in defining different subtypes of disease [3,4]. The WHO classification was updated in 2008, further reinforcing the integration of these four elements in the diagnosis of lymphoma [5].

The WHO lymphoma classification is now well known and widely used by hematopathologists, making the approach to diagnosis more consistent. However, lymphomas still remain a diagnostic challenge among general pathologists. The literature on this topic reveals that widely varying agreement values have been reported recently [6,7,8,9,10]. These studies encompassed short periods and/or assessed relatively small numbers of cases. Furthermore, a vast majority of these studies included case samples from 2008 and before; therefore, information regarding the situation for more recent years is not known. We thus designed our study to investigate the situation in Turkey with many more cases to cover a longer period.

The İstanbul University Cerrahpaşa Faculty of Medicine (CFM) Hematopathology Service is a reference center receiving specimens from several hospitals. In order to test the validity of the hypothesis that adoption of the WHO classification by pathologists resulted in less discrepancy among centers in correctly diagnosing lymphoma, we carried out a retrospective study by reviewing all lymphoma or lymphoma-suspected specimens that had been sent to our laboratory for a second opinion between 2000 and 2013.

## MATERIALS AND METHODS

All specimens that had been referred to the CFM between 2000 and 2013 (excluding those with cutaneous biopsies only) for a second opinion were deemed eligible if the records of the original biopsy results were available.

Biopsy specimens with a definite or suspected initial diagnosis of lymphoma were reevaluated at the CFM by an expert in hematopathology (N.T.). Initial diagnoses were not considered discordant if they defined the lymphoma type correctly but failed to give additional features related to grade (e.g., follicular lymphoma grades 1 to 2) or subtype [e.g., germinal center vs. activated B-cell types of diffuse large B-cell lymphoma (DLBCL)]. Divergent diagnoses among subtypes of T-cell lymphomas were not considered discordant since they would only minimally affect the clinical approach. 

During the course of this study, 206 benign samples were received. These typically were cases in which the primary pathologist could not definitively rule out lymphoma or cases in which the patient had a history of lymphoma and displayed suggestive clinical features.

To evaluate whether diagnostic discrepancy had an effect on the clinical management of the patients, we reviewed the discordant samples and confined them into one of three groups according to the differences between the referral and revised diagnoses (Table 1). Cases were grouped depending on whether the revisions would alter treatment and management according to the National Comprehensive Cancer Network guidelines, as previously described [6,11,12].

Cases where the primary pathologist or second opinion failed to reach a definitive diagnosis were also included in the study and classified as non-diagnostic. A case that was initially diagnosed as non-diagnostic was included in group B if it received a benign diagnosis upon second opinion and in group C if it received a malignant diagnosis, since it caused a delay in the commencement of therapy. Cases classified as non-diagnostic after a second opinion were considered neither concordant nor discordant and were not included in statistical analysis.

To evaluate the change in the discrepancy rates of lymphoma diagnosis over time we compared the rates of revision between 2000-2008 (group 1) and 2009-2013 (group 2). Specimens from 1 January 2000 to 31 December 2008 (group 1) and from 1 January 2009 to 31 December 2013 (group 2) were evaluated using the WHO 2001 and 2008 classifications, respectively. However, our purpose in doing so was not to compare the two WHO classifications, which are essentially very similar, but rather to assess the adoption of the WHO classification by general pathologists over time.

Statistical analysis was done using SPSS 15.0 for Windows. The comparison of the diagnostic revision rates was carried out using chi-square or Fisher’s exact tests.

## RESULTS

A total of 1824 patients in two time periods (1008 between 2000 and 2008 and 816 between 2009 and 2013) met the inclusion criteria and were assessed. A definite diagnosis could not be attributed to 126 cases after a second opinion due to various reasons. These cases were not included in the statistical analysis. Analyses were conducted based on 1698 cases that had a definitive diagnosis following a second opinion.

Initially 1372 patients had an initial diagnosis of one of the lymphoid malignancies. This number increased to 1450 after revision at the CFM. All cases diagnosed as lymphoma after a second opinion are listed together with the initial diagnoses in Table 2A and 2B.

The majority of group A was composed of lymphoma typing discrepancies in both periods (Table 3). Even with the improved concordance rate in histological subtypes over time, the histological subtypes that frequently mimic these diagnoses were generally similar. DLBCLs, the most common diagnosis, were frequently misdiagnosed as classical Hodgkin lymphoma (cHL) (n=24) in both periods. All of those cases were T-cell rich B-cell lymphoma (TCRBCL), a subtype of DLBCL. cHL, the second most common diagnosis, was frequently misdiagnosed as T-cell lymphoma (TCL) (n=11) in both periods. The majority of those cases were the anaplastic large cell lymphoma (ALCL) subtype. Nodular lymphocyte-predominant Hodgkin lymphoma (NLPHL) was frequently misdiagnosed as cHL (n=11) in both periods. While TCL cases were frequently misdiagnosed as cHL, with 10 such cases in the first period, this situation was completely improved in the second period (n=0). In the second period, the most common histologic type, revised as TCL, was TCRBCL with two cases.

The majority of group B was composed of typing deficiency of the low-grade B-cell lymphoma group (Table 3). Fifty-two of the 57 cases diagnosed as low-grade lymphoma not otherwise specified (LL-NOS) were revised as lymphoma.

The majority of group C, including changes that may lead to delay in treatment, was composed of ambiguous diagnoses. Of the 129 cases of unspecified lymphoma (L-NOS) as initial diagnosis, 109 were revised as lymphoma. After expert review, the majority of cases were reclassified as DLBCL (n=49).

There were some cases called atypical lymphoid infiltration (ALI) that did not specify a fully benign or malignant diagnosis. Fifty-six and 61 such cases were received in the first and second periods, respectively. Thirty-seven and 34 of those cases were identified as lymphoma after expert review, respectively. While cHL (n=12) was the most diagnosed subtype in the first period, cHL (n=8) and DLBCL (n=8) were equal in the second period. The majority of the remaining cases were classified as benign diagnoses (n=25). After review, 2 cases were reclassified as non-lymphoid lesions: granulocytic sarcoma and histiocytosis.

Thirty-three and 18 cases in the first and second periods had been received with a diagnosis of undifferentiated malignant tumor (UMT), respectively. Only one of the cases was also diagnosed as UMT after expert review. Twenty-eight of the remaining cases were reclassified as lymphoma after expert review in the first period. The most frequently diagnosed histological subtype was DLBCL (n=17). After revision there were also two non-lymphoma diagnoses, which were lymphoma-like lesion of the cervix (n=1) and granulocytic sarcoma (n=1). For the remaining two cases, it was inappropriate to make a diagnosis with the given materials. All of the cases (n=18) in the second period were classified as lymphoma after revision. The most frequently diagnosed lymphoma subtype was DLBCL (n=11).

Twenty-nine samples had an initial diagnosis of lymphoma, which was changed to benign/reactive. These were 20 cases of reactive hyperplasia (RH), 2 thymoma, 1 necrosis, 2 Kikuchi’s disease, 2 progressive transformation of germinal centers (PTGC), 1 Castleman’s disease, and 1 lymphoepithelial sialadenitis (LESA).

Of the 20 cases diagnosed as RH at our center, 1 case was called ALCL at the referring center. This lesion was developed after a purified protein derivative test. One case was called Burkitt lymphoma (BL), but this lesion had occurred after bee stings. Other cases were plasma cell neoplasia (PCN) (n=1), L-NOS (n=4), MCL (n=1), NLPHL (n=1), high grade B-cell lymphoma (HL-NOS) (n=1), FL G2 (n=1), FL G1 (n=2), cHL (n=5), and unspecified B-cell lymphoma (B-NHL) (n=2). Of the 2 cases diagnosed as thymoma at our center, one was assigned as B-NHL and the other TCL at the referring centers. Of the two cases diagnosed as Kikuchi’s disease at our center, one was assigned as L-NOS and the other as cHL. Of the two cases diagnosed as PTGC at our center, one was assigned as FL G2 and the other as NLPHL. The case diagnosed as Castleman’s disease at our center was assigned as LL-NOS. The case diagnosed as necrosis at our center was assigned as B-NHL. The case diagnosed as LESA at our center was assigned as HL-NOS at the referring center.

Of the 206 samples with an initial diagnosis of a reactive or benign condition, 37 were changed to lymphoid malignancy after expert review (Table 2A and 2B).

Fourteen samples had an initial diagnosis of lymphoma, which was changed to a non-lymphoid diagnosis. Nine of the cases in the first period and 5 of the cases in the second period were sent with a histologic type of lymphoma diagnosis. Of the 3 cases with a referral diagnosis of cHL, 2 were reclassified as carcinoma and 1 as histiocytosis. Two cases had a referral diagnosis of HL-NOS, where 1 was reclassified as carcinoma and the other as granulocytic sarcoma. Four cases with the initial diagnosis of L-NOS were reclassified, 2 as carcinoma and 1 each as granulocytic sarcoma and choriocarcinoma. There were 3 cases with a referral diagnosis of TCL, and 1 had the diagnosis revised to thymoma, 1 was reclassified as nasopharyngeal carcinoma, and 1 was reclassified as small cell lung carcinoma (it was sent with a diagnosis of “NK cell leukemia/lymphoma”). One case with a referral diagnosis of B-NHL was reclassified as thymoma. One case with a referral diagnosis of DLBCL was reclassified as nasopharyngeal carcinoma.

Twenty-one cases with a primary diagnosis of non-lymphoid malignancy were defined as lymphoma after a second opinion. The most common malignancy mimicking lymphoma was poorly differentiated/undifferentiated carcinoma.

The overall discordance rate was 45.6% (774 of 1698 samples). This rate showed significant variations between different histologic subtypes. In 343 of the 774 patients with discordant diagnoses, a second review would lead to a considerable change in the clinical management of the patient (group A). In 114 patients the revised result would have only minimal impact on the patient care (group B), while in 316 patients the insufficient primary diagnoses would lead to delayed or potentially inappropriate treatment without the second opinion review (group C).

Discordance rates also varied substantially over time. The overall discordance rate decreased from 51.3% in 2000-2008 to 38.7% in 2009-2013 (p<0.0001). The discordance rate in group A decreased from 24.7% to 14.7% (p<0.0001). Changes in other categories (groups B and C) were not statistically significant (Table 4).

In this study, 189 relapsed cases were also sent for consultation. A definite diagnosis could not be attributed to 15 of these cases with the given materials. In these relapsed cases, major changes (32.8%, n=62, p<0.0001) and overall discrepancy rate (59.8%, n=98, p=0.1) were higher when compared to the overall study.

There was a higher rate of major revision in diagnoses from non-academic centers (257/1142, 22.5%) compared to academic centers (63/302, 20.9%). However, the rates were not significantly different (p=0.59).

## DISCUSSION

This article reports the experience at the CFM with second-opinion pathology review, showing an overall concordance rate of 54.4%. Compared to studies in other regions, the discordance rate of this study is higher, especially compared to Western countries where rates of less than 20%-30% have been recorded [6,7,8]. Some of the most important reasons for this are probably the recent initiation of widespread use of immunohistochemistry (IHC), deficiencies in selection of the right IHC staining panels, evaluation and implementation in primary centers, and the lack of an official training system for hematopathology in Turkey. The study that is most similar to ours in terms of selection of cases, by Matasar et al. in 2006, reported a major revision rate of 18.6% [6], and Chang et al. reported a rate of 55% [9]. In our study, we found revision rates that could change clinical management (groups A and C) as 44.2% in the first period and 31.5% in the second.

Clinically meaningful discrepancies for every subtype of lymphoma were seen and varied considerably between lymphoma subtypes. Surprisingly, the rate of discordance of the most common subtype in Turkey, DLBCL, was high.

TCLs are relatively rare in our geographic area and this prevents pathologists from gaining experience related to this entity. Although we grouped all mature TCLs into one category and excluded cutaneous lymphomas, there was still a high discordance rate. As indicated in the study of Herrera et al. [13], current and future therapeutic approaches target subsets of TCLs, and accurate diagnosis and distinguishing between TCL subtypes promises to become even more important. This suggests the necessity of getting a second opinion from an expert hematopathologist in cases of TCLs.

It was seen that grading of follicular lymphoma, and especially of FL G3, is still difficult for many pathologists, despite being one of the more common subtypes of lymphoma. This suggests the necessity of getting a second opinion from an expert hematopathologist in at least the grading of FL, and, in our opinion, in cases of low-grade lymphoma unclassified.

In our analysis, we found a surprisingly high discordance rate for mantle cell lymphomas. Because of the availability of ancillary tests such as cyclin D1 in mantle cell lymphoma, it can be considered an “easy” diagnosis. However, the pathologist must recognize certain features in histopathology in order to use this ancillary test. A retrospective look revealed that 7 out of 40 discordant cases did not have IHC utilized in the initial diagnosis, and this may be one of the factors decreasing the concordance.

One major concern is that we found a higher rate of major discrepancies in relapsed cases. Unfortunately, the majority of these patients had received treatment for a while before being sent for a second opinion. Therefore, it can be said that these patients were treated with an inappropriate regimen for a while.

There are some limitations to our study. First, the second review was performed by one expert pathologist. Another limitation of our study is that the pathologist was not blinded to the initial diagnoses.

In conclusion, in countries where widespread use of ancillary techniques like IHC and fluorescent in situ hybridization by general pathologists is a recent development, and therefore the effect of WHO classification is newly starting to be seen, the level of discordance is greater. Despite this, rates were still high in the second period, which may be caused by technical insufficiency and incorrect evaluation of IHC. The higher rate of diagnostic divergence especially in the second period indicates the need for easily accessible hematopathology consultation centers, and based on our results, we would advocate that a hematopathology fellowship education system be established.

## Figures and Tables

**Table 1:**
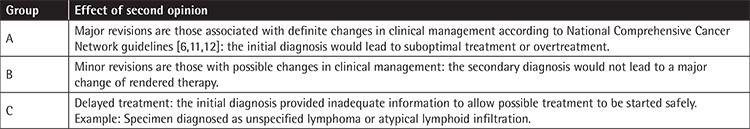
Grouping of discrepant diagnoses according to their effect on treatment.

**Table 2A:**
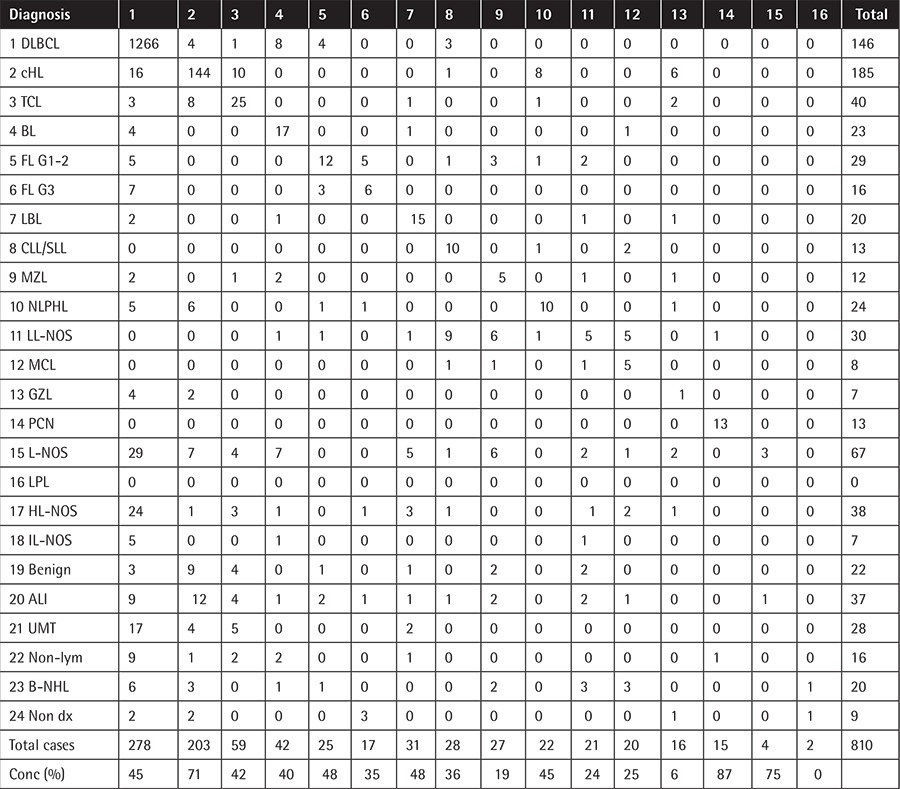
Referral and final pathologic diagnoses in 2000-2008 (n=810).

**Table 2B:**
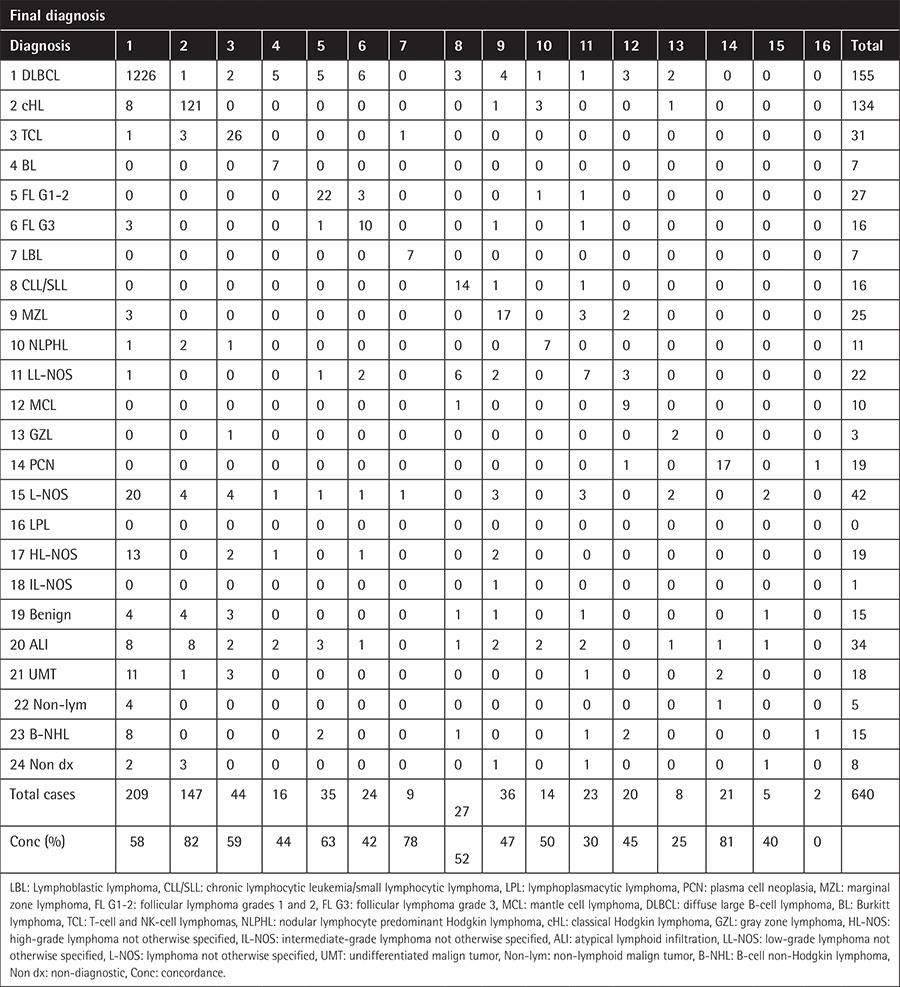
Referral and final pathologic diagnoses in 2009-2013 (n=640).

**Table 3:**
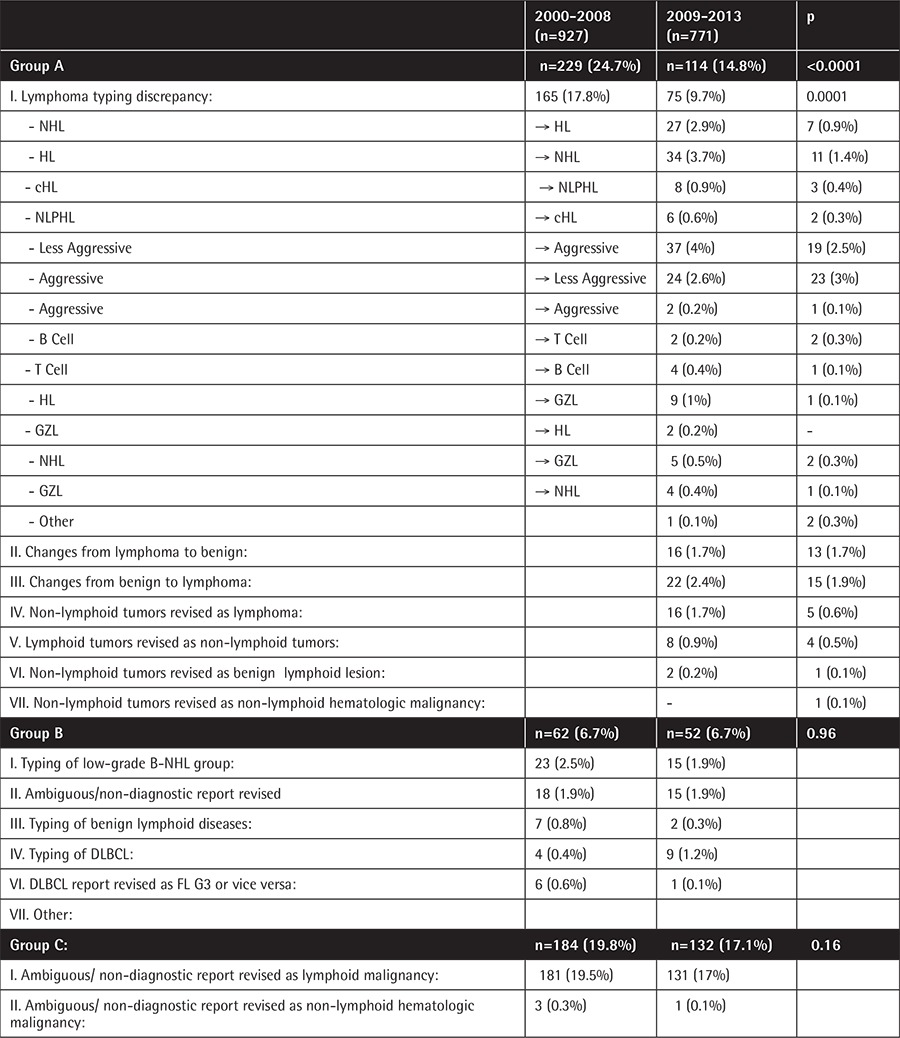
Summary of the diagnostic discrepancies in lymphoma diagnosis by category in 2000-2008 and 2009-2013.

**Table 4:**
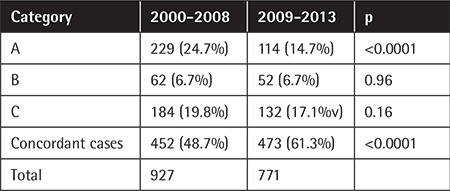
Comparison of distributions of diagnostic revision, 2000-2008 and 2009-2013.
